# Repetition Without Repetition: Challenges in Understanding Behavioral Flexibility in Motor Skill

**DOI:** 10.3389/fpsyg.2020.02018

**Published:** 2020-08-13

**Authors:** Rajiv Ranganathan, Mei-Hua Lee, Karl M. Newell

**Affiliations:** ^1^Department of Kinesiology, Michigan State University, East Lansing, MI, United States; ^2^Department of Kinesiology, University of Georgia, Athens, GA, United States

**Keywords:** synergy, variability, motor learning, coordination, skill acquisition

## Abstract

A hallmark of skilled motor performance is behavioral flexibility – i.e., experts can not only produce a movement pattern to reliably and efficiently achieve a given task outcome, but also possess the ability to change that movement pattern to fit a new context. In this perspective article, we briefly highlight the factors that are critical to understanding behavioral flexibility, and its connection to movement variability, stability, and learning. We then address how practice strategies should be developed from a motor learning standpoint to enhance behavioral flexibility. Finally, we highlight some important future avenues of work that are needed to advance our understanding of behavioral flexibility. We use examples from sport as a context to highlight these issues, especially in regard to elite performance and development.

## Introduction

A fundamental hallmark of motor skill is “behavioral flexibility” – i.e., skilled performers are not only consistent and efficient at producing goal-directed behavior, but also have the ability to do so even in altered conditions or environments (Johnson, 1961). For example, the “grand slam” in tennis is considered one of the highest achievements in the sport because it requires winning on vastly different surfaces that require flexibility in playing style. Although the central concept of behavioral flexibility in motor control has been recognized since Bernstein’s use of the phrase “repetition without repetition” to describe how even well-learned movements show variation when achieving the task outcome (Bernstein, 1967), there are only a few studies that directly examine this issue in the context of skilled performance (Arutyunyan et al., 1969; Bootsma and van Wieringen, 1990; Cohen and Sternad, 2009). Furthermore, we still have a limited understanding of its relation to other constructs such as learning, development, and operational aspects such as practice strategies. The focus of this perspective article is not to present or examine a specific theoretical position, but instead to highlight open theoretical and practical issues surrounding behavioral flexibility and suggest directions for future work.

## Challenge 1: Characterizing Behavioral Flexibility

Behavioral flexibility is a broad term that has been used in several contexts and can often overlap with other terms such as transfer or generalization. In this article, we focus specifically on behavioral flexibility in terms of the ability to achieve the *same task outcome* using different movement solutions (as opposed to transfer/generalization which often refer to achieving *novel task outcomes*). A related term that has been used in this context is “adaptability” – which shares features with flexibility (Seifert et al., 2014), but we will use the term flexibility because adaptability has also been used in a broader sense to indicate better generalization to new environments (Seidler et al., 2015). Given the focus on task outcomes, we will examine flexibility within the same skill domain (e.g., within the same sport) and not across domains. An important condition for such flexibility is the presence of degeneracy (Edelman and Gally, 2001) – sometimes also called redundancy (Bernstein, 1967) or abundance (Latash, 2012; although these terms are not always interchangeable) – where structurally different elements can be coordinated in different ways to produce the same task outcome. For example, if the desired task outcome in tennis to land the ball at a particular point on the court, flexibility refers to the ability to use multiple movement solutions that achieve this task outcome. These multiple solutions include solutions that can be termed variations of the same movement pattern (e.g., using a forehand with different amounts of topspin) and solutions that are distinct enough to be termed “qualitatively” different (e.g., using a backhand or a running volley). Given this definition, we highlight three important factors that can be used to characterize behavioral flexibility in the context of motor skill.

### Flexibility Can Occur Over Different Time Scales

The timescale over which the transition(s) between the old and new movement solutions occur is a critical aspect of flexibility (Newell et al., 2001). Flexibility may be observed over relatively short-time scales (of the order of a few seconds) on a trial-to-trial basis, as seen in the classic study of expert blacksmiths (Bernstein, 1967). But flexibility can also be observed over longer time scales requiring relearning of a new movement pattern or implementation of another movement technique or strategy (Wallis et al., 2002; Napier et al., 2015; Gray, 2018). The time scale of change also relates behavioral flexibility to the related construct of “stability” (Schöner and Kelso, 1988). Although formal definitions of stability are not directly related to variability (van Emmerik and van Wegen, 2000; Dingwell and Marin, 2006), the term stability in motor skill has been used to refer to consistency. This stability can be present at two levels – “task-level” stability (measured by variability of the task outcome) and a “movement-level” stability (measured by movement variability). Flexibility at short time scales (e.g., at the level of trial to trial variability) is associated with high task-level stability and (relatively) low movement-level stability so that multiple movement solutions can be used. However, flexibility at longer time scales (e.g., modifying someone’s technique) involves *increasing* movement-level stability of the new solution so that the performer does not return to the old solution. For example, an athlete who has changed their throwing technique after injury would not want to return to their “old” movement pattern even if they could achieve the task outcome using the old solution.

### Flexibility Can Be Explicit or Implicit

It is important to consider the degree of change involved in generating the new movement solution because this directly ties into how the flexibility is generated – either through explicit “strategy-like” behavior (Taylor and Ivry, 2011; Christensen and Bicknell, 2019; Christensen et al., 2019) or through implicit “synergy-like” behavior. Strategic changes are likely associated with cognitive skills, such as anticipation and decision-making, and involve relatively large modifications to the movement patterns that could be employed in contexts, where there is a distinct change in the environment (e.g., adjusting to different surfaces in tennis). Additionally, strategic changes may also arise when there is a need to surprise an opponent (e.g., a between-the-legs shot in tennis). On the other hand, when the desired change is minimal, flexibility can be achieved by channeling the natural movement variability (i.e., variability observed in the task without any externally imposed perturbations) through “synergies” that constrain the degrees of freedom. For example, there is evidence that expert shooters are able to reduce the variability at the hand by employing a compensatory coordination between the shoulder and wrist movement (Arutyunyan et al., 1969). These synergies are likely created through extensive practice and do not require strategic behavior.

### Flexibility Can Arise From Different Constraints

From a dynamical framework, identifying the source of the constraint that induces the need for new movement solutions is important to understand behavioral flexibility. Constraints at the individual (organism), task, and environmental levels can all lead the performer to adopt different movement solutions (Higgins, 1977; Newell, 1986). However, the dynamics of the available solution space and how it is perceived by the performer depends to a large extent on the source of the constraint. Task and environmental constraints (e.g., a change in playing conditions) can change over short time scales and are typically large enough to be apparent to the performer and, therefore, provide a window into strategy-like flexibility. On the other hand, most individual constraints change gradually over relatively long time scales (e.g., fatigue) or very long time scales (e.g., growth; Newell et al., 2001) and, therefore, are a window into more synergy-like flexibility.

## Challenge 2: Linking Movement Variability, Flexibility, and Task Performance

**Figure 1:**
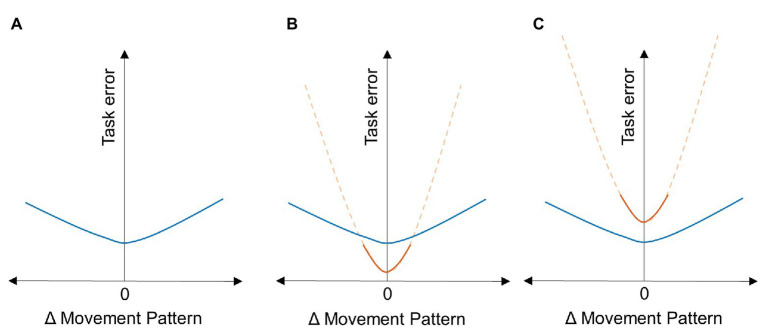
Flexibility, movement variability, and task performance. The plots indicate potential relations between the task error and the change from a preferred movement pattern. **(A)** Flexibility can be measured at an individual level by measuring the “flatness” of this curve (indicated in blue). An individual with greater flexibility will have a flatter curve indicating that they are capable of using multiple movement solutions to achieve (approximately) the same task error. However, comparisons between-individuals provide greater insight into whether this flexibility comes at a cost. An individual with lesser flexibility (indicated in red) is shown with a smaller range of movement variability (solid line) and the extrapolation of this curve to the same range as the more flexible individual (dashed line). The relative positions of these two individuals on the task error axis can reveal the potential cost of flexibility. **(B)** If the more flexible individual has higher task error, then flexibility comes at the cost of lower task performance. **(C)** However, if the more flexible individual has lower task error, then it suggests that greater flexibility can potentially lead to finding solutions with higher task performance.

The amount of movement variability and its relation to task performance is the central focus of behavioral flexibility and is closely related to “stability.” By the definition assumed here, an individual with greater flexibility should be able to generate the same task performance with greater changes in the movement pattern relative to an individual with less flexibility. Thus, on a plot of the change in the movement pattern vs. task error, flexibility can be measured by how “shallow” this curve is (Figure 1A). However, our view is that this “within-person” measurement alone does not provide the whole picture of flexibility because it ignores the issue of whether this flexibility comes at a cost. For example, when now comparing two individuals (or equivalently two groups) with different degrees of flexibility, greater flexibility may result in higher task error (Figure 1B), which would be suggestive of a trade-off between movement-level stability and flexibility. On the other hand, greater flexibility may result in lower task error (Figure 1C), which would be consistent with the notion that the variation associated with flexible behaviors may help the performer to find new solutions that optimize task performance even further. Therefore, both within‐ and between-individual analyses are necessary to gain a full understanding of how flexibility affects task performance.

In addition to the amount of movement variability, it is also important to consider the structure of variability (Newell and Slifkin, 1998). This structure can give insight into “exploration” – both in terms of how multiple degrees of freedom are involved in the movement and how these behaviors evolve over time. This distinction between the amount and structure of variability is critical from the viewpoint of characterizing exploration. For example, it is well-established that children show higher motor variability overall relative to adults in a wide range of tasks (Deutsch and Newell, 2001). However, when the structure of this variability was examined when children learned a novel task, children actually showed less exploration relative to adults because they expressed that variability mostly along a single coordination pattern (Lee et al., 2018). Similarly, sequential analysis of trial-to-trial behavior has emphasized that the variation when exploring is not typically “random” but shows specific patterns of exploration from trial-to-trial depending on the context (Dingwell et al., 2013). Overall, these findings suggest that the relation between movement variability and flexibility is complex and mediated by several factors. This becomes especially relevant during learning when the amount of movement variability, the structure of movement variability, and task performance all change with practice.

## Challenge 3: Enhancing Flexibility Through Practice Strategies

How can behavioral flexibility be enhanced through practice strategies – i.e., how can we structure practice so that the learner learns to use multiple movement solutions to achieve a given task outcome? We highlight two broad but distinct “routes” to increase flexibility through practice – direct and emergent, each with several theoretical orientations.

### Direct Flexibility Elicited During Practice

The first approach to enhance flexibility is to directly practice multiple movement solutions for achieving a given task outcome (Ranganathan and Newell, 2013). From a “specificity of practice” interpretation, if the flexibility to use multiple solutions is desired, then these multiple solutions have to be practiced. Even assuming a certain degree of transfer beyond the practiced solutions, a key aspect of this approach is to introduce variation during practice to elicit new movement solutions. This has been addressed in a number of theoretical frameworks, but is particularly prominent in the dynamical systems framework, where these variations are interpreted as fluctuations that can help transition from one solution to another. Two related approaches inspired by this framework have been suggested in the literature – the nonlinear pedagogy approach (Chow et al., 2011), which emphasizes the use of appropriate constraints to facilitate these transitions, and the differential learning approach, which emphasizes the amplification of fluctuations inherent in the learner (Schöllhorn et al., 2012). However, to date, studies using these approaches have focused mainly on improving overall task performance, so it remains to be seen if they also apply to enhancing behavioral flexibility.

### Emergent Flexibility After Practice

The second approach takes a somewhat counterintuitive notion that increasing flexibility need not require multiple solutions to be *directly* practiced, but rather flexibility is an “emergent” feature with learning. In other words, flexibility is not the primary goal but rather a *by-product* of training. This is particularly relevant for open skills such as tennis or soccer (Poulton, 1957), where the unpredictable nature of the environment constantly requires coming up with novel solutions in both short and long time scales that cannot be directly practiced.

One such example of emergent flexibility comes from optimal feedback control, where flexible ways of achieving the task outcome can emerge because rather than choose a solution *a priori*, the system constantly looks for a solution that minimizes both error and effort to achieve the task outcome. For example, in an obstacle avoidance task (Nashed et al., 2014), where participants had to navigate around multiple obstacles, flexibility in behavior for reaching the same target (either going between obstacles or going around them) was observed depending both on the magnitude of the perturbation and the estimated position of the hand (i.e., the sensory feedback). Similarly, when examining learning a target interception task with different obstacle positions, we found that participants who practiced without variation but learned the target position well could adapt to different obstacles, even if they had not explicitly practiced with different obstacle positions (Ranganathan and Newell, 2010). These results suggest that flexibility, at least when the degree of variation is small, can emerge without direct practice of different solutions.

This emergent flexibility can also be seen with the ability to perceive the appropriate affordances. A famous example of extreme behavioral flexibility involves Gael Monfils’ “spinning jump forehand,” where he ran back from the net to return a lob, and then performed a spinning jump to return a forehand winner (Dawson, 2019). It is rather unlikely that he would have practiced this shot to any significant degree during training. Rather it was the ability to pick up the appropriate information (the time to contact with the ball, but also the higher bounce on clay) that enabled a “creative” solution to emerge under novel constraints without direct practice (Orth et al., 2017).

Finally, because perception and action are interrelated (Gibson, 1979), the ability to pick up affordances is also intricately tied with the movement repertoire of the individual. Many of the examples of behavioral flexibility described above are only feasible because of the athlete’s characteristics – such as strength, speed, and joint range of motion. Therefore, another possibility to increase behavioral flexibility is to increase this movement repertoire during training. This effectively would increase the degeneracy of the system so that more flexible behaviors are possible.

## Avenues for Future Research

We highlight three avenues to further our knowledge of behavioral flexibility – (i) the measurement of flexibility in motor learning designs, (ii) characterizing behavioral flexibility over development, and (iii) better understanding the constraints on behavioral flexibility at elite (or near-elite) performance levels.

### Measurement of Flexibility in Motor Learning Designs

A primary limitation of current motor learning studies in terms of studying behavioral flexibility is the combination of simple laboratory tasks and the exclusive reliance on retention/transfer tests. The use of “richer” tasks, where there are possibilities of multiple solutions either at the individual (multiple DOFs) or the task/environment, is essential to gain insight into flexibility (Newell, 1991; Ranganathan and Scheidt, 2016; Sternad, 2018).

How could behavioral flexibility be measured in such tasks? Two different approaches can be used to provide a complementary understanding of both explicit and implicit flexibility during motor learning. The first approach is to use quantitative methods for analyzing movement variability (Scholz and Schöner, 1999; Cohen and Sternad, 2009). These techniques provide insight into how natural variability is channeled in the task with no external perturbations and, therefore, are a good window into implicit flexibility with small magnitudes of change. However, it is important to note that there is a risk in these techniques of using “observed” variability to infer the flexibility. This is because (i) the relation between observed variability and flexibility is likely non-monotonic (i.e., too much or too little variability can both be “bad”; Stergiou et al., 2006) and (ii) unless measured in a context that requires flexibility, the observed variability tends to typically decrease with practice, even though flexibility may have increased (Ranganathan and Newell, 2010). Therefore, a second approach is to directly change the constraints to challenge the learner’s flexibility and observe how well the task outcome is met (Ranganathan and Newell, 2010; Komar et al., 2015; Orth et al., 2019). This approach overcomes the disadvantage of using observed flexibility as a metric, however, because the learner is generally aware of a change in these constraints, it is therefore better suited to study explicit flexibility involving larger changes in the movement pattern.

### Flexibility Over Developmental Timescales

Development over the life span provides an opportunity to understand the influence of individual constraints on behavioral flexibility. Development is characterized by both physical changes (e.g., growth during childhood or the loss of muscle mass in old age) and cognitive changes (e.g., working memory and information processing capacity), and there is at least some evidence that flexibility and exploration during motor learning change over the life span (Lee et al., 2018; Lee and Ranganathan, 2019). This gives rise to important questions like – how does behavioral flexibility develop with age and how does it relate to other aspects of development? Moreover, this also has important implications for how practice strategies should be tailored to developmental age and skill level. Currently, the main approach behind tailoring practice strategies relies on setting an appropriate level of task difficulty (Guadagnoli and Lee, 2004). However, understanding how flexibility (and stability) changes with development would have direct real-world relevance to issues such as the emphasis on consistency and variability during practice (Whiteside et al., 2015). More broadly, this issue also relates to the role of early specialization vs. diversification in the development of expertise in sport skills (Côté et al., 2009), specifically related to the issue of when it might be appropriate to start diversification without disrupting the desired skill.

### Flexibility at Elite Levels of Performance and Technique Modification

From the viewpoint of elite (or near-elite) performance levels, it is important to recognize that different sports skills have differing demands for behavioral flexibility. For example, in closed skills like gymnastics, behavioral flexibility may not be as critical given the relatively predictable nature of the environment. However, in open skills like tennis or soccer, where the constantly varying environment places high demands on behavioral flexibility, there are two issues that need to be addressed – (i) how flexibility changes with high levels of performance and (ii) how flexibility plays a role in the specific context of technique modification.

First, at high levels of performance, there are two mutually competing demands on flexibility. On the one hand, at elite performance levels, there is *less* room for flexibility because the space of possible solutions is considerably narrowed. For example, there are fewer movement patterns to hit a forehand at 80 mph compared to hitting a forehand at 50 mph. On the other hand, at higher levels of performance, there is a need for *more* flexibility because of constraints such as the need to adapt to different surfaces, game strategies, and the need to deceive opponents by being more unpredictable. Therefore, understanding how high-level performers manage these competing demands on flexibility, and the analysis of individual differences at these levels (Dicks et al., 2010; Müller et al., 2015), is an important avenue for future work.

Second, an extremely relevant topic related to elite athletes and flexibility is the issue of “technique modification” – i.e., reorganizing from an existing movement solution to a new movement solution (Napier et al., 2015; Gray, 2018). Although there are plenty of examples of elite players changing their movement pattern to improve performance or reduce injury, there is very little information available on the process of how this reorganization occurs. Anecdotally, evidence during such technique modification is characterized by lower levels of performance for rather sustained periods of time (weeks to months) before reaching pre-modification levels. This pattern is consistent with a dynamical systems view that long-term flexibility does not occur on a blank slate, and that the stability of prior patterns has an influence on how easy it is to be flexible. In particular, finding ways to experimentally address issues of multistability and metastability (Kelso, 2012) in motor learning (Hristovski et al., 2006; Liu et al., 2010; Pinder et al., 2012) may be critical to understanding technique modification and can provide insight into how practice strategies may be developed to accelerate relearning in the presence of prior solutions. This understanding will not only have implications for athletes, but also for movement rehabilitation, where movement patterns have to be modified in the context of a prior pattern to achieve the same goal (Ranganathan, 2017).

## Concluding Comments

**Figure 2:**
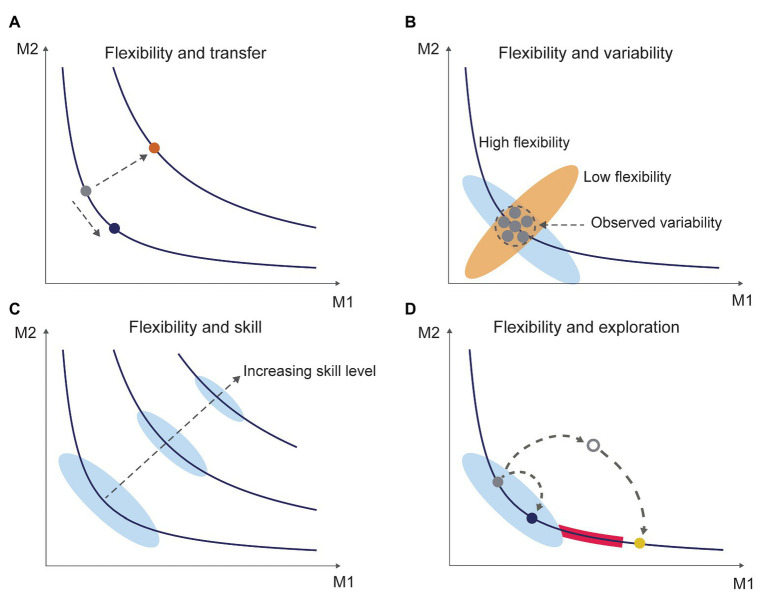
Flexibility and associated constructs of learning. Each plot shows two hypothetical movement parameters (M1 and M2). Contours represent combinations of movement parameters that achieve the same task outcome, and so each point on a given contour represents a “movement solution” to achieve that task outcome (Latash et al., 2002). **(A)** Flexibility and transfer. Flexibility in the current definition refers to moving from a point on the contour to another point on the *same* contour (i.e., same task outcome, indicated in blue). Transfer on the other hand refers to moving from a point on the contour to a *different* contour (i.e., different task outcome, indicated in orange). **(B)** Flexibility and variability. Inferring flexibility directly from the observed movement variability can be difficult because the observed variability in movement patterns (shown inside the circle, with each dot representing a different trial) could either be a part of a movement repertoire with high (blue ellipse) or low flexibility (red ellipse). **(C)** Flexibility and skill. As skill levels and associated task performance levels go up, the degeneracy available in the system (shown by the ellipses) generally goes down. This makes it a challenge to find new solutions to achieve the same task outcome at high skill levels. **(D)** Flexibility and exploration. Exploration refers to the process of finding a new movement solution. Exploration can be quick when solutions are within the same movement pattern (indicated in blue). However, there may be regions on the contour that are unstable (indicated by the red band), which require prolonged exploration and creativity to find a qualitatively different movement solution (indicated in yellow).

Classic definitions of motor skill emphasize aspects such as task achievement, consistency, and efficiency (Guthrie, 1952), yet behavioral flexibility is critical to understand how these aspects emerge in dynamically changing contexts. Behavioral flexibility intersects with several central themes in motor behavior such as variability, learning, and practice strategies and provides a fertile ground for future work. The issues raised here (summarized in Figure 2) provide a basis for a renewed focus on behavioral flexibility that go beyond Bernstein’s (Bernstein, 1967) original observation, and we anticipate that this will lead to theoretical and practical advances in a wide range of domains.

## Data Availability Statement

The original contributions presented in the study are included in the article/supplementary material, further inquiries can be directed to the corresponding author.

## Author Contributions

RR, M-HL, and KN contributed to the conceptualization of the idea. RR and M-HL designed the visualizations. RR wrote the first draft of the manuscript. RR, M-HL, and KN edited and revised the manuscript. All authors contributed to the article and approved the submitted version.

### Conflict of Interest

The authors declare that the research was conducted in the absence of any commercial or financial relationships that could be construed as a potential conflict of interest.
